# Prostate cancer risk: associations with ultraviolet radiation, tyrosinase and melanocortin-1 receptor genotypes

**DOI:** 10.1054/bjoc.2001.2097

**Published:** 2001-11

**Authors:** C J Luscombe, M E French, S Liu, M F Saxby, P W Jones, A A Fryer, R C Strange

**Affiliations:** Department of Urology, North Staffordshire Hospital, Staffordshire; Department of Mathematics, Keele University, Staffordshire; Clinical Biochemistry Research Laboratory, School of Postgraduate Medicine, Keele University, North Staffordshire Hospital, Staffordshire, ST4 7PA, UK

**Keywords:** prostate cancer, susceptibility, polymorphism, ultraviolet radiation

## Abstract

Exposure to ultraviolet radiation may reduce prostate cancer risk, suggesting that polymorphism in genes that mediate host pigmentation will be associated with susceptibility to this cancer. We studied 210 prostate cancer cases and 155 controls to determine whether vitamin D receptor (VDR, Taql and Fokl variants), tyrosinase (TYR, codon 192 variant) and melanocortin-1 receptor (MC1R, Arg151Cys, Arg160Trp, Val92Met, Asp294His and Asp84Glu variants) genotypes are associated with risk. UV exposure was determined using a questionnaire. MC1R Arg^160^/Arg^160^ homozygotes were at increased risk (*P* = 0.027, odds ratio = 1.94) while TYR *A2*/*A2* homozygotes were at reduced risk of prostate cancer (*P* = 0.033, odds ratio = 0.48). These associations remained significant after correction for UV-exposure. Stratification of cases and controls by quartiles of exposure, showed that the protective effect of TYR A1A2 (*P* = 0.006, odds ratio 0.075) and A2A2 (*P* = 0.003, odds ratio 0.055) was particularly strong in subjects who had received the greatest exposure. Our data show for the first time, that allelism in genes linked with skin pigment synthesis is associated with prostate cancer risk possibly because it mediates the protective effects of UV. Importantly, susceptibility is associated with an interaction between host predisposition and exposure. © 2001 Cancer Research Campaign  http://www.bjcancer.com


*British Journal of Cancer* (2001) **85**(10), 1504–1509
© 2001 Cancer Research Campaign
doi: 10.1054/ bjoc.2001.2097, available online at http://www.idealibrary.com on IDEAL

http://www.bjcancer.com

### Prostate cancer risk: associations with ultraviolet radiation, tyrosinase and melanocortin-1 receptor genotypes

**CJ Luscombe[1], ME French[1], S Liu[1], MF Saxby[1], PW Jones[2], AA Fryer[3] and RC Strange[3]**

[1]Department of Urology, North Staffordshire Hospital, Staffordshire; [2]Department of Mathematics, Keele University, Staffordshire; and [3]Clinical Biochemistry Research Laboratory, School of Postgraduate Medicine, Keele University, North Staffordshire Hospital, Staffordshire, ST4 7PA, UK

**Summary** Exposure to ultraviolet radiation may reduce prostate cancer risk, suggesting that polymorphism in genes that mediate host pigmentation will be associated with susceptibility to this cancer. We studied 210 prostate cancer cases and 155 controls to determine whether vitamin D receptor (VDR, Taql and Fokl variants), tyrosinase (TYR, codon 192 variant) and melanocortin-1 receptor (MC1R, Arg151Cys, Arg160Trp, Val92Met, Asp294His and Asp84Glu variants) genotypes are associated with risk. UV exposure was determined using a questionnaire. MC1R $Arg^{160}/Arg^{160}$ homozygotes were at increased risk ($P$ = 0.027, odds ratio = 1.94) while TYR *A2/A2* homozygotes were at reduced risk of prostate cancer ($P$ = 0.033, odds ratio = 0.48). These associations remained significant after correction for UV-exposure. Stratification of cases and controls by quartiles of exposure, showed that the protective effect of TYR A1A2 ($P$ = 0.006, odds ratio 0.075) and A2A2 ($P$ = 0.003, odds ratio 0.055) was particularly strong in subjects who had received the greatest exposure. Our data show for the first time, that allelism in genes linked with skin pigment synthesis is associated with prostate cancer risk possibly because it mediates the protective effects of UV. Importantly, susceptibility is associated with an interaction between host predisposition and exposure. © 2001 Cancer Research Campaign http://www.bjcancer.com

**Keywords**: prostate cancer; susceptibility; polymorphism; ultraviolet radiation

Exposure to ultraviolet radiation (UV) has multiple effects in skin. These include deleterious events that are linked with increased skin cancer risk (Kricker et al, 1994; Kripke, 1994) as well as beneficial effects that include, according to several studies, reduced risk of other cancers including those in colon, breast and ovary (Blair and Fraumeni, 1978; Garland et al, 1980, 1990; Studzinski and Moore, 1995; Ekman et al, 1997; Selby and Mawer, 1999; McCarty, 2000). In this context, North American-based studies linking latitude with prostate cancer mortality, have been interpreted as showing that UV has a protective effect on development of this cancer (Hanchette and Schwartz, 1992). Indeed, the authors argued that the link of risk with latitude could not be explained by other factors such as diet.

The mechanism whereby UV might influence prostate cancer risk is unclear though both vitamin D and parathyroid hormone have been implicated (Studzinski and Moore, 1995; Selby and Mawer, 1999; McCarty, 2000). Thus, 1,25 $(OH)_2$ vitamin D has effects on tumour cell proliferation, differentiation and spread and, parathyroid hormone acts as a tumour promoter (Studzinski and Moore, 1995; Haussler et al, 1998; McCarty, 2000). Indeed, UV exposure may explain why subclinical rather than malignant prostate disease is very common in older men. Thus, blood-borne factors, whose concentration is determined by UV, could ensure that tumours remain latent rather than progress to malignancy (Hanchette and Schwartz, 1992). The concentration of such factors will in part, be determined by skin colour and therefore, individual ability to initiate pigment synthesis may mediate the harmful and beneficial effects of exposure (Sturm et al, 1998; Rees and Healy, 1997). In particular, the synthesis of melanin is worthy of study as it largely determines skin colour. The first rate-limiting steps of melanin synthesis, hydroxylation of tyrosine to 3,4-dihydroxy-phenylalanine and its dehydrogenation to dopaquinone are catalyzed by tyrosinase (TYR) under the influence of melanocyte stimulating homone (Sturm et al, 1998; Oetting and King, 1999). This hormone acts via the melanocortin-1 receptor (MC1R). Both genes demonstrate polymorphisms with functional consequences; some mutations of TYR cause albinism (Oetting and King, 1999) and MC1R variants are associated with hair colour and skin type (Valverde et al, 1995; Rees and Healy, 1997; Box et al, 1997; Abel-Malek et al, 1999; Palmer et al, 2000). Vitamin D is also important. Its metabolites are formed in skin following UV exposure and can promote differentiation and inhibit proliferation of prostate cancer cells (Haussler et al, 1998). Synthesis of such metabolites will fall with increased pigmentation. Further, several studies have shown associations between vitamin D receptor (VDR) genotypes and prostate cancer risk (Taylor et al, 1996; Ingles et al, 1997).

We now describe studies in prostate cancer cases and benign prostatic hypertrophy (BPH) controls to determine whether disease risk is mediated by polymorphism in VDR (*Taql* and *Fokl* variants), TYR (codon 192 variants) and MC1R (Arg151Cys, Arg160Trp, Val92Met, Asp294His, Asp84Glu alleles). The influence of these genes may depend on the amount of UV exposure received. Accordingly, we also assessed acute and chronic exposure using a questionnaire that has been independently validated (Harvey et al, 1996a, 1996b; Ramsay et al, 2000).

*Received 4 April 2001*
*Accepted 1 August 2001*

*Correspondence to:* RC Strange

### MATERIALS AND METHODS

### Patients

We recruited, between October 1999 and May 2000, 210 prostate cancer cases and 155 BPH controls. They were unrelated, Northern European Caucasians from urology clinics in the North Staffordshire Hospital. Because definitive diagnosis of prostate cancer and BPH can be difficult, we established inclusion and exclusion criteria for these groups. Histological evidence of prostatic adenocarcinoma was obtained in 190 patients. In 20 severely ill cases with clinically obvious prostate cancer, it was considered unethical to subject patients to the potential morbidity of a biopsy. These cases had a clinically malignant prostate gland on digital rectal examination, positive bone scan and prostatic specific antigen levels above 30 ng/ml (age-related reference range up to 6.5 ng/ml). The proportions of cancer cases with well, moderately and poorly differentiated tumours (WHO grade) was as expected. Similarly, using the TNM staging system, local T stage and metastatic state at presentation were typical. BPH patients had a serum prostatic specific antigen level in the age-related reference range and a benign digital rectal examination. Histological confirmation of BPH was obtained in 123 subjects. The North Staffordshire Hospital Ethics Committee approved the study and informed consent was obtained from all patients. The BPH comparison group was chosen because the diagnosis of BPH largely allows exclusion of concurrent prostate cancer.

### Assessment of UV exposure

All subjects completed a validated questionnaire (Harvey et al, 1996a, 1996b; Ramsay et al, 2000) that recorded; (i) Childhood sunburning, defined as erythema for more than 48 h or blistering. This parameter was scored as yes/no and the number of recalled sunburning events was recorded. (ii) Adult sunbathing score calculated as follows; the extent of sunbathing was recorded as never, rarely, occasionally and frequently (scored 1, 2, 3 and 4 units respectively) in three age categories (20–39 years, 40–59 years and above 60 years). The score was obtained by adding the units from the three age categories. (iii) History of regular foreign holidays in a sunny country with mean weeks/year. (iv) Daily sun exposure (weekdays and weekends considered separately in the three age categories shown above and combined to give total cumulative exposure). (v) Proportion of working life spent outdoors. (vi) History of residence in a hot country for more than 6 months. Skin type (Ramsay et al, 2000), hair and eye colour were recorded.

### Genotype identification

DNA was extracted using phenol-chloroform from 5 ml blood. The A→C change in exon 1 (codon 192) of TYR was identified as described (Giebel and Spritz, 1990). PCR-RFLP assays were used to identify VDR alleles with exon 2 (Fok1) and exon 9 (Taq1) variants (Hutchinson et al, 2000). MC1R Arg151Cys and Arg160Trp variants were detected using primers 5′-TCTCCATCTTCTACG-CATTG-3′ and 5′-GCCAGCATGTGGACGTACAG-3′ to amplify a 202 bp product. PCR conditions were 95˚C (2 min) and 35 cycles of 95˚C (1 min), 55˚C (1 min), 72˚C (1 min), followed by 72˚C (3 min). Arg151Cys was identified by digestion with BsrDl (65˚C, 18 h) and products were examined after electrophoresis in 2% agarose. $Arg^{151}/Arg^{151}$ and $Cys^{151}/Cys^{151}$ subjects had fragments of 180 bp and 22 bp and, 202 bp respectively. Arg160Trp was identified by digestion with Sacll (37˚C, 18 h), and products examined in 3% agarose. $Arg^{160}/Arg^{160}$ and $Trp^{160}/Trp^{160}$ subjects had fragments of 156 bp and 46 bp and, 202 bp. Val92Met, Asp294His and Asp84Glu variants were identified as described (Ichii-Jones et al, 1998).

### Statistical methods

Data was analysed using Stata version 6 for Windows (State Corporation, College Station, TX). All tests of significance were two sided. Student's *t*-test was used to demonstrate an age difference and logistic regression was used to compare frequencies of parameters in cases and controls. Step-wise logistic regression, with a threshold for inclusion/exclusion of a variable of $P = 0.1$, was used to identify the best set of predictors. We recognize that examination of eight genotypes for links with phenotype risks identification of false associations. However, correction for multiple testing may increase the risk of type II errors (Perneger, 1998). Accordingly, we present uncorrected *P*-values but recognize our exploratory findings require confirmation in another case group. This approach minimizes loss of true positive results but allows false positive results to be identified (Perneger, 1998; Cuzick, 1999).

### RESULTS

### VDR, TYR and MC1R genotypes and prostate cancer risk

A complete genotype data set was obtained from all but 2 subjects (1 from each group). These DNA samples were refractory to amplification in all the PCR assays. Logistic regression analysis was used to identify associations between the eight individual polymorphisms and susceptibility to prostate cancer (Table 1). As the mean age at diagnosis of cases (70.6 years) was significantly greater ($P < 0.001$) than that of controls (67.0 years), we included correction for this imbalance in all logistic regression models. For the MC1R loci, we compared frequencies of homozygotes for the wild type allele against combined frequencies of heterozygotes and homozygotes for the mutant allele (Table 1). This approach was used because mutant allele frequencies are generally small and, heterozygotes as well as homozygotes for mutant alleles are associated with sun-sensitive phenotypes (13). VDR and TYR genotypes were factorized as shown (Table 1). We first analysed each gene individually, correcting only for age at diagnosis in the model. Six polymorphisms showed no significant association with prostate cancer risk (data not shown). However, MC1R $Arg^{160}/Arg^{160}$ homozygotes were at increased risk ($P = 0.027$, odds ratio = 1.94, 95% CI = 1.08–3.49) while TYR A2/A2 homozygotes were at reduced risk ($P = 0.033$, odds ratio = 0.48, 95% CI = 0.25–0.94).

We next determined whether the associations of individual genotypes with risk were influenced by inclusion of the parameters of UV exposure derived from the questionnaire. These were; history of childhood sunburning, adult sunbathing score, history of regular foreign holidays, history of living in a sunny country for > 6 months, ratio of occupational time spent indoors/outdoors, skin type, hair and eye colour. We also studied cumulative UV exposure. In this context, unpublished studies in these cases and controls

© 2001 Cancer Research Campaign

**Table 1** Comparison of genotype frequencies in cases and controls

| | Controls (%) | Cases (%) | *P*-value | Odds ratio | 95% CI |
|---|---|---|---|---|---|
| MC1R | | | | | |
| $Arg^{151}/Arg^{151}$ | 104 (67.5) | 152 (72.7) | 0.356 | 1.29 | 0.75–2.24 |
| $Arg^{151}/Cys^{151}$ and $Cys^{151}/Cys^{151}$ | 50 (32.5) | 57 (27.3) | reference | 1 | N/A |
| $Arg^{160}/Arg^{160}$ | 122 (79.2) | 184 (88.0) | 0.023 | 2.14 | 1.11–4.12 |
| $Arg^{160}/Trp^{160}$ and $Trp^{160}/Trp^{160}$ | 32 (20.8) | 25 (12.0) | reference | 1 | N/A |
| $Val^{92}/Val^{92}$ | 129 (83.8) | 170 (81.3) | 0.523 | 0.82 | 0.43–1.55 |
| $Val^{92}/Met^{92}$ and $Met^{92}/Met^{92}$ | 25 (16.2) | 39 (18.7) | reference | 1 | N/A |
| $Asp^{294}/Asp^{294}$ | 144 (93.5) | 194 (92.8) | 0.587 | 0.70 | 0.19–2.56 |
| $Asp^{294}/His^{294}$ and $His^{294}/His^{294}$ | 10 ( 6.5) | 15 ( 7.3) | reference | 1 | N/A |
| $Asp^{84}/Asp^{84}$ | 148 (96.1) | 202 (96.7) | 0.374 | 0.64 | 0.24–1.70 |
| $Asp^{84}/Glu^{84}$ and $Glu^{84}/Glu^{84}$ | 6 ( 3.9) | 7 ( 3.3) | reference | 1 | N/A |
| Tyrosinase | | | | | |
| A1A1 | 18 (11.7) | 41 (19.6) | reference | 1 | N/A |
| A1A2 | 71 (46.1) | 101 (48.3) | 0.245 | 0.65 | 0.31–1.35 |
| A2A2 | 65 (42.2) | 67 (32.1) | 0.033 | 0.44 | 0.21–0.94 |
| VDR | | | | | |
| FF | 65 (42.2) | 85 (40.7) | reference | 1 | N/A |
| Ff | 65 (42.2) | 92 (44.0) | 0.775 | 0.93 | 0.55–1.57 |
| ff | 24 (15.6) | 32 (15.3) | 0.505 | 0.78 | 0.37–1.63 |
| TT | 57 (37.0) | 70 (33.5) | reference | 1 | N/A |
| Tt | 67 (43.5) | 110 (52.6) | 0.996 | 1.00 | 0.57–1.73 |
| tt | 30 (19.5) | 29 (13.9) | 0.266 | 0.66 | 0.32–1.40 |

Logistic regression analysis was used to compare the frequencies of each individual genotype in a model that included age at diagnosis, childhood sunburning, adult sunbathing score, positive holiday history, proportion of occupation spent indoor/outdoor, living abroad for more than 6 months, skin type, eye and hair colour and UV exposure in the lowest quartile.

showed that cumulative exposure in the lowest quartile (less than 1639 days of lifetime exposure) was a better predictor of prostate cancer risk than total cumulative exposure (Luscombe et al, 2001). Accordingly, in further logistic regression models, we included sun exposure in the lowest quartile instead of total cumulative exposure. MC1R $Arg^{160}/Arg^{160}$ and TYR A2/A2 remained significantly associated with cancer risk (Table 1).

We further confirmed these associations by using stepwise logistic regression analysis to identify the best set of predictors of prostate cancer in the presence of other variables. The model included all parameters derived from the questionnaire and the eight genotypes and age at diagnosis. Table 2 shows that four UV predictors and, MC1R $Arg^{160}/Arg^{160}$ and TYR A2/A2 were all significantly associated with prostate cancer risk. Childhood sunburning, on the basis of its odds ratio (0.13) had the greatest influence. The values of the odds ratio for the genotypes were similar to those of the other UV predictors suggesting equal impact.

### Genotype associations in patients stratified by exposure

We next determined whether the association of TYR A1A2 and A2A2 with cancer risk was more evident in cases stratified into sub-groups based on quartiles of total cumulative exposure. We found that in 46 BPH controls whose cumulative exposure was in the highest quartile, 44 subjects were TYR A1A2 or A2A2 (95.7%). In the corresponding 47 prostate cancer cases, 32 subjects were TYR A1A2 or A2A2 (68.1%). Logistic regression analysis demonstrated that these proportions were significantly different (TYR A1A2, $P = 0.006$, *A2A2* $P = 0.003$, Table 3) even after correction for the UV parameters found to be significant in the step-wise routine (childhood sunburning, adult sunbathing score and holidays abroad). By contrast, the frequencies of these genotypes were not significantly different ($P = 0.98$, $P = 0.45$, respectively) in 162 cases and 108 controls whose exposure was in the first, second or third quartiles. Table 3 shows that the association of MC1R $Arg^{160}/Arg^{160}$ with increased risk was found in patients from both quartiles 1–3 and 4 though the link only achieved significance in subjects with exposure in quartiles 1–3.

### DISCUSSION

The causes of prostate cancer are unclear though data in twins shows that environmental factors account for over 50% of risk (Lichtenstein et al, 2000). However, the impact on risk, of suggested causative factors including diet and occupation are relatively modest (odds ration < 2.5) (Ekman et al, 1997). Latitude and by inference, UV exposure have also been implicated (Blair and Fraumeni, 1978; Hanchette and Schwartz, 1992). To further explore this hypothesis we speculated that genes associated with host response to UV would be associated with susceptibility to this cancer. We used BPH patients as controls because the condition appears part of normal ageing and is not associated with increased prostate cancer risk (Young et al, 2000). Further, diagnosis of BPH, requires exclusion of prostate cancer and as screening for this cancer is not advised in the United Kingdom, BPH patients are the only available group that is unlikely to have this malignancy. Quantification of UV exposure is difficult as data is often collected decades after exposure events from elderly patients. It is however, essential if the effects of UV on cancer risk are to be assessed. Various questionnaires

 *© 2001 Cancer Research Campaign*

**Table 2** Stepwise logistic regression analysis to identify the best set of predictors for prostate cancer risk

| Predictor | *P*-value | Odds ratio | 95% CI |
|---|---|---|---|
| Childhood sunburning | < 0.001 | 0.13 | 0.06–0.31 |
| Age at diagnosis | < 0.001 | 1.07/year | 1.03–1.10 |
| Adult sunbathing score | < 0.001 | 0.84/unit | 0.77–0.91 |
| Foreign holidays | 0.003 | 0.44 | 0.26–0.76 |
| Lowest 25% of cumulative exposure | 0.005 | 2.37 | 1.30–4.34 |
| MC1R $Arg^{160}/Arg^{160}$ | 0.014 | 2.24 | 1.18–4.24 |
| Tyrosinase: A2A2 | 0.037 | 0.50 | 0.36–0.97 |

The stepwise model included: history of childhood sunburning, age at diagnosis, adult sunbathing score, history of regular foreign holidays, Lowest 25% of cumulative exposure, history of living in a sunny country for > 6 months, ratio of occupational time spent indoors and outdoors, hair colour, eye colour and skin type. The following genotypes were also included: MC1R; Arg151Cys ($Arg^{151}/Arg^{151}$ vs $Arg^{151}/Cys^{151}$ and $Cys^{151}/Cys^{151}$), Arg160Trp ($Arg^{160}/Arg^{160}$ vs $Arg^{160}/Trp^{151}$ and $Trp^{151}/Trp^{151}$), Val92Met ($Val^{92}/Val^{92}$ vs $Val^{92}/Met^{92}$ and $Met^{92}/Met^{92}$), Asp294His ($Asp^{294}/Asp^{294}$ vs $Asp^{294}/His^{294}$ and $His^{294}/His^{294}$), Asp84Glu ($Asp^{84}/Asp^{84}$ vs $Asp^{84}/Glu^{84}$ and $Glu^{84}/Glu^{84}$), TYR A2A2 (A1 as reference) and VDR; Fok I (FF as reference) and Taq I (TT as reference).

**Table 3** Association of TYR genotypes with prostate cancer risk in cases stratified by UV exposure

| | *P*-value | Odds ratio | 95% CI |
|---|---|---|---|
| **TYR genotypes** | | | |
| (i) association of *TYR* genotypes with prostate cancer risk in the 93 cases and BPH controls who received the highest quartile of UV exposure: | | | |
| TYR A1A2 | 0.006 | 0.075 | 0.011–0.48 |
| TYR A2A2 | 0.003 | 0.055 | 0.008–0.37 |
| Age at diagnosis | 0.698 | 1.01/year | 0.94–1.09 |
| Holidays in hot climates | 0.837 | 1.12 | 0.35–3.71 |
| Childhood sun burning | 0.027 | 0.039 | 0.002–0.69 |
| Adult sunbathing score | < 0.001 | 0.68/unit | 0.56–0.83 |
| (ii) association of *TYR* genotypes with prostate cancer risk in the 270 cases and BPH controls who received UV exposure in quartiles 1–3: | | | |
| TYR A1A2 | 0.98 | 0.99 | 0.44–2.20 |
| TYR A2A2 | 0.47 | 0.73 | 0.32–1.67 |
| Age at diagnosis | < 0.001 | 1.07/year | 1.03–1.11 |
| Holidays in hot climates | 0.005 | 0.41 | 0.22–0.77 |
| Childhood sun burning | 0.027 | 0.17 | 0.07–0.42 |
| Adult sunbathing score | < 0.001 | 0.88/unit | 0.79–0.97 |
| **MC1R genotypes** | | | |
| (i) association of *MC1R* genotypes with prostate cancer risk in the 93 cases and BPH controls who received the highest UV exposure (quartile 4): | | | |
| MC1R $Arg^{160}/Arg^{160}$ | 0.33 | 1.95 | 0.51–7.47 |
| Age at diagnosis | 0.52 | 1.02/year | 0.95–1.10 |
| Holidays in hot climates | 0.76 | 0.84 | 0.28–2.52 |
| Childhood sun burning | 0.02 | 0.072 | 0.008–0.66 |
| Adult sunbathing score | < 0.001 | 0.72/unit | 0.61–0.85 |
| (ii) association of MC1R genotypes with prostate cancer risk in the 270 cases and BPH controls who received UV exposure in quartiles 1–3: | | | |
| MC1R $Arg^{160}/Arg^{160}$ | 0.029 | 2.26 | 1.08–4.67 |
| Age at diagnosis | 0.001 | 1.06/year | 1.03–1.10 |
| Holidays in hot climates | 0.002 | 0.37 | 0.20–0.70 |
| Childhood sun burning | < 0.001 | 0.16 | 0.06–0.39 |
| Adult sunbathing score | 0.015 | 0.88/unit | 0.80–0.98 |

The data show a comparison of the frequencies of genotypes and UV-derived parameters in prostate cancer cases and BPH controls using logistic regression analysis. The model included the parameters shown.

have been used to assess exposure. We used a questionnaire that has been validated in independent studies in skin cancer patients (Harvey et al, 1996a,b) and, renal transplant recipients (Ramsay et al, 2000).

The VDR, TYR and MC1R genes have a presumed role in host response to UV. We recognize that in highly allelic genes, selection of alleles for study is problematical. In this regard, we selected variants on the basis of presumed function and frequency. Clearly other alleles at these genes could demonstrate strong associations with prostate cancer risk though in many cases their frequencies are small. Each of the alleles studied is therefore, regarded as a marker on chromosome 12q (VDR), 11q (TYR) or 16q (MC1R) that could be in linkage disequilibrium with the true candidate gene. Further, we reported associations between individual genotypes and susceptibility and cannot infer that particular genotypes define an individual response to UV. Indeed, some subjects classified as homozygotes for wild type alleles may be homozygous or compound heterozygous for variant alleles not included in our series. It is noteworthy however, that we found associations between MC1R genotypes and skin type. Thus, the frequencies of MC1R $Arg^{160}/Arg^{160}$,

© 2001 Cancer Research Campaign 

Arg$^{151}$/Arg$^{151}$ and Asp$^{84}$/Asp$^{84}$ in cases and controls increased through skin types 1– 4 (data not shown). By contrast, Val$^{92}$/Val$^{92}$ and Asp$^{294}$/Asp$^{294}$ were not associated with skin type. Since only four controls and two cases had red hair, it was not possible to assess whether MC1R genotypes were associated with hair colour.

We studied two of the five polymorphic sites identified in the VDR. Allelism at the Fok1 site alters the upstream start codon resulting in production of a shortened protein from the F allele (Haussler et al, 1998). The Taq1 restriction site is in linkage disequilibrium with three other 3′-UTR polymorphisms; *Bsm1*, *Apa1* and a poly(A) tail microsatellite. These variants may also be functionally significant because the length of the poly(A) repeat affects mRNA stability. TYR is highly allelic with more than 90 alleles identified (Oetting and King, 1999). We studied the allele resulting from an A→C change in exon 1. While this change has not been shown to have functional consequences, the A1 and A2 alleles have similar frequencies (0.44 and 0.56 in cases) and are useful markers. The MC1R is also highly polymorphic with more than 20 alleles identified (Sturm et al, 1998; Rees and Healy, 1997; Valverde et al, 1995; Palmer et al, 2000; Box et al, 1997; Abel-Malek et al, 1999). We studied Arg151Cys, Arg160Trp and Asp294His, which are linked with red hair, Val92Met which has been linked with skin type and Asp84Glu which has been associated with malignant melanoma risk in one but not other studies (Sturm et al, 1998; Rees and Healy, 1997; Valverde et al, 1995; Palmer et al, 2000; Box et al, 1997; Abel-Malek et al, 1999; Ichii-Jones et al, 1998).

We found that only MC1R Arg$^{160}$/Arg$^{160}$ and TYR A2A2 were significantly associated with prostate cancer risk. These analyses were performed with individual genes and, with and without, inclusion of UV parameters in the logistic regression models. Homozygosity for MC1R Arg$^{160}$ was associated with increased risk (odds ratio = 2.18) while homozygosity for TYR A2 allele was linked with reduced risk of cancer (odds ratio = 0.42). Neither the other MC1R genotypes nor the VDR sites were linked with risk. A stepwise routine that included all genotypes and parameters from the UV questionnaire confirmed these findings. While there are no published data on associations between TYR or MC1R genotypes and prostate cancer risk, links between VDR Taq1 variants have been reported (odds ratio for tt 0.34) (Taylor et al, 1996). We found that VDR tt was linked with lower risk (odds ratio 0.66) than TT though the difference was not significant ($P$ = 0.252). The reason for this difference is unclear but may reflect differences in the relative impact of environmental and genetic factors in these populations. We next determined whether associations between genotypes and prostate cancer risk were dependent on UV exposure. We found that the protective effect of TYR genotypes, found in the total group, reflects an association with risk in subjects with the highest quartile of exposure. Though the functional consequences of the polymorphism are unknown, we speculate that the variant allele is associated with less efficient production of melanin and reduced pigmentation. Thus, the beneficial effects of high UV exposure will be more effectively transmitted systemically. It is unclear why this link is not found in subjects who received less UV exposure. By contrast the link of MC1R Arg$^{160}$/Arg$^{160}$ with risk was found in subjects with exposure in quartiles 1–3 and 4 though the association in subjects with greatest exposure was not significant. No associations between VDR genotypes and risk were found in these groups.

Polymorphism in various genes including VDR, androgen receptor and prostatic specific antigen, has been linked with prostate cancer susceptibility (Taylor et al, 1996; Ingles et al, 1997; Xue et al, 2000). Our data show for the first time, that allelism in genes linked with skin pigment synthesis is associated with risk. We recognize that while UV appears critical in the development of skin cancers, its role in determining susceptibility to prostate cancer is speculative (Studzinski and Moore, 1995). Nonetheless, while our data are preliminary and require confirmation in a separate case group, they demonstrate some internal consistency. Thus, aspects of chronic and acute UV exposure and, genes associated with response to UV were associated with risk. Some associations demonstrated small $P$ values and significance would be observed even after correction for multiple testing using a rigorous approach such as that described by Bonferroni (Perneger, 1998).

### ACKNOWLEDGEMENTS

We gratefully acknowledge the generous support of the staff of Wedgwood Plc, Barlaston, Staffordshire, England.

### REFERENCES

Abel-Malek Z, Suzuki I, Tada A, Im S and Akcali C (1999) The melanocortin-1 receptor and human pigmentation. *Ann NY Acad Sci* **885**: 117–133

Blair A and Fraumeni JF (1978) Geographic patterns of prostate cancer in the United States. *J Natl Cancer Inst* **61**: 379–384

Box NF, Wyeth JR, O'Gorman LE, Martin NG and Sturm RA (1997) Characterization of melanocyte stimulating hormone receptor variant alleles in twins with red hair. *Human Mol Genet* **6**: 1891–1897

Cuzick J (1999) Interaction, subgroup analysis and sample size. In: *Metabolic polymorphisms and cancer,* Boffetta P, Caporaso N, Cuzick J, Lang M and Vineis P (eds) pp 109–121; No 148. Lyon: IARC Scientific Publications

Ekman P, Pan Y, Li C and Dich J (1997) Environmental and genetic factors: a possible link with prostate cancer. *Br J Urol* **79**: **Suppl 2**:35–41

Garland CF and Garland FC (1980) Do sunlight and vitamin D reduce the likelihood of colon cancer? *Int J Epidemiol* **9**: 227–231

Garland FC, Garland CF, Gorham ED and Young JF (1990) Geographic variation in breast cancer mortality in the United States: a hypothesis involving exposure to solar radiation. *Prevent Med* **19**: 614–622

Giebel LB and Spritz RA (1990) RFLP for *Mbo1* in the human tyrosine (TYR) gene detected by PCR. *Nucl Acid Res* **18**: 3103

Hanchette CL and Schwartz GG (1992) Geographic patterns of prostate cancer mortality. Evidence for a protective effect of ultraviolet radiation. *Cancer* **70**: 2861–2869

Harvey I, Frankel S, Marks R, Shalom D and Nolan-Farell M (1996a) Non-melanoma skin cancer and solar keratoses. 1. Methods and descriptive results of the South Wales skin cancer study. *Br J Cancer* **74**: 1302–1307

Harvey I, Frankel S, Marks R, Shalom D and Nolan-Farell M (1996b) Non-melanoma skin cancer and solar keratoses. 2. analytical results of the South Wales skin cancer study. *Br J Cancer* **74**: 1308–1312

Haussler MR, Whitfield GK, Haussler CA, Hsieh JC, Thompson PD, Selznick SH and Whitfield GK (1998) The nuclear vitamin D receptor biological and molecular regulatory properties revealed. *J Bone Miner Res* **13**: 325–349

Hutchinson PE, Osborne JF, Lear JT, Smith AG, Bowers B, Morris PN, Jones PW, York C, Strange RC and Fryer AA (2000) Vitamin D receptor polymorphisms are associated with altered prognosis in patients with malignant melanoma. *Clin Cancer Res* **6**: 498–504

Ichii-Jones F, Lear JT, Heagerty AHM, Smith AG, Hutchinson PE, Osborne J, Bowers B, Jones PW, Davies E, Ollier WER, Thomson W, Yengi L, Bath J, Fryer AA and Strange RC (1998) Susceptibility to malignant melanoma: Influence of skin type and polymorphism in the melanocyte stimulating hormone receptor gene. *J Invest Dermatol* **111**: 218–221

Ingles SA, Ross RK, Yu MC, Irvine RA, La Pera G, Haile RW and Coetzee GA (1997) Association of prostate cancer risk with genetic polymorphisms in vitamin D receptor and androgen receptor. *J Natl Cancer Inst* **89**: 166–170

Kricker A, Armstrong BK and English DR (1994) Sun exposure and non-melanocytic skin cancer. *Cancer Causes Control* **5**: 367–392

Kripke ML (1994) Ultraviolet radiation and immunology: Something new under the Sun. *Cancer Res* **54**: 6102–6105

 *© 2001 Cancer Research Campaign*

Lichtenstein P, Holm NV, Verkasalo PK, lliadou A, Kaprio J, Koskenvuo M, Pukkala E, Skytthe A and Hemminki K (2000) Environmental and heritable factors in the causation of cancer. *New Engl J Med* **343**: 78–85.

Luscombe CJ, Fryer AA, French ME, Liu S, Saxby MF, Jones PW and Strange RC (2001) Exposure to ultraviolet radiation: association with susceptibility and age at presentation with prostate cancer. *Lancet* **358**: 641–642

McCarty MF (2000) Parathyroid hormone may be a cancer promoter- an explanation for the decrease in cancer risk associated with ultraviolet light, calcium and vitamin D. *Med Hypoth* **54**: 475–482

Oetting WS and King RA (1999) Molecular basis of albinism: Mutations and polymorphisms of pigmentation genes associated with albinism. *Hum Mutat* **13**: 99–115

Palmer JS, Duffy DL, Box NF, Aitken JF, O'Gorman LE, Green AC, Haywood NK, Martin NG and Sturm RA (2000) Melanocortin-1 receptor polymorphisms and risk of melanoma: Is the association explained solely by pigmentation phenotype? *Am J Hum Genet* **66**: 176–186

Perneger TV (1998) What's wrong with Bonferroni adjustments. *BMJ* **316**: 1236–1238

Ramsay HM, Fryer AA, Reece S, Smith AG and Harden PN (2000) Clinical risk factors associated with non-melanoma skin cancer in renal transplant recipients. *Am J Kidney Dis* **36**: 167–176

Rees JI and Healy E (1997) Melanocortin receptors, red hair, and skin cancer. *J Invest Dermatol Symposium Proc* **2**: 94–98

Selby PL and Mawer EB (1999) Exposure to sunlight may reduce cancer risk. *BMJ* **319**: 1066

Studzinski GP and Moore DC (1995) Sunlight– Can it prevent as well as cause cancer? *Cancer Res* **55**: 4014–4022

Sturm RA, Box NF and Ramsay M (1998) Human pigmentation genetics: the difference is only skin deep. *Bioessays* **20**: 1–11

Taylor JA, Hirvonen A, Watson M, Pittman G, Mohler JL and Bell DA (1996) Association of prostate cancer with vitamin D receptor gene polymorphism. *Cancer Res* **56**: 4108–4110

Valverde P, Healy E, Jackson I, Rees JL and Thody AJ (1995) Variants of the melanocyte-stimulating hormone receptor gene are associated with red hair and fair skin in humans. *Nature Genet* **11**: 328–330

Xue W, Irvine RA, Yu MC, Ross RK, Coetzee GA and Ingles SA (2000) Susceptibility to prostate cancer: interaction between genotypes at the androgen receptor and prostate specific antigen. *Cancer Res* **60**: 839–841

Young JM, Muscatello DJ and Ward JE (2000) Are men with lower urinary tract symptoms at increased risk of prostate cancer? A systematic review and critique of the available evidence. *Br J Urol Int* **85**: 1037–1048

*© 2001 Cancer Research Campaign*
